# An Empirically Informed Analysis of the Ethical Issues Surrounding Split Liver Transplantation in the United Kingdom

**DOI:** 10.1017/S0963180116000086

**Published:** 2016-07

**Authors:** GREG MOORLOCK, JAMES NEUBERGER, SIMON BRAMHALL, HEATHER DRAPER

**Keywords:** split liver transplantation, transplant staff, liver transplant patients, liver transplantation

## Abstract

Surgical advances have allowed for the development of split liver transplantation, providing two recipients with the opportunity to potentially benefit from one donated liver by splitting the liver into two usable parts. Although current data suggest that the splitting of livers provides overall benefit to the liver-recipient population, relatively low numbers of livers are actually split in the United Kingdom. This article addresses the question of whether ethical concerns are posing an unnecessary barrier to further increasing the number of life-saving transplantations. Recognizing that an important aspect of exploring these concerns is gaining insight into how transplant staff and patients regard splitting livers, the article presents the findings of a qualitative study examining the views of senior transplant staff and liver transplant patients in the UK and uses these to inform a commentary on the ethical issues relating to split liver transplantation.

## Background

This article uses qualitative data gathered from staff and patients at UK liver transplant centers to inform a commentary on the ethical issues raised by the UK transplant community’s use of split livers. Liver transplantation in the UK faces a familiar problem: the high demand for liver transplants, combined with a relatively low supply of transplantable livers,^1^ has meant that patients needing transplants are placed on waiting lists and often endure long periods of illness before a liver suitable for transplant becomes available. At the time of writing, there are more than 500 patients waiting for a liver transplant in the UK,^2^ and the median waiting time for an adult liver transplant patient is 147 days.^3^ Some patients will die on the waiting list or will become too ill to benefit from a transplant and will be removed from the waiting list.^4^ In the past, this shortage of livers, although it affected all potential transplant recipients, was a particular problem for the pediatric population. Liver size in relation to potential recipient size is an important factor when matching livers to recipients. A whole liver from an adult is normally too large for a child,^5^ and pediatric donation is rare. Before livers were split, average waiting times and waiting list mortality for pediatric patients were particularly high.^6^

Surgical advances have allowed for the development of split liver transplantation, which provides two recipients with the opportunity to potentially benefit from one donated liver by splitting the liver into two usable parts. The two recipients of a split liver are usually an adult and a child, although sometimes the recipients are an adult and a smaller-sized adult. Splitting livers has reduced waiting times and subsequent waiting list mortality for children,^7^ because parts of livers that could otherwise have been given whole to adults are “diverted” to the pediatric population.

Splitting a liver is a complicated surgical procedure, as is the subsequent transplant, which can present specific technical challenges, such as maintaining sufficient blood supply and biliary drainage.^8^ These challenges have historically been reflected in worse outcomes for recipients of split livers (split livers are regarded as marginal grafts),^9^ which has given rise to complex ethical issues. Initial results with split livers were mixed, which led to reluctance on the part of transplant centers to fully embrace the approach. Splitting livers is now, however, an established procedure in the UK, and the Liver Advisory Group of National Health Service Blood and Transplant (NHSBT) provides guidelines for compulsory consideration of splitting of livers that meet specific criteria. Outcomes of split liver transplantation have improved over time but vary among transplant centers, and some data suggest that the adult recipient in particular is still at a higher risk of posttransplant complications than if he or she received a whole liver.^10^ To mitigate these risks, only the best-quality livers are split (these are livers from donors under 40 years who have died following brainstem death and who have had short ICU stays). This raises the question of whether it is preferable to provide a larger number of transplants and accept that the risks associated with these may be greater or to focus on providing fewer but lower-risk liver transplants. Because children are better off and adults are worse off as a result of splitting livers, it has been suggested that the practice privileges the interests of children, which may be a form of age discrimination. There are also concerns about exposing adults to increased risks that they did not fully understand when making the decision to accept a transplant.

Current data suggest that the splitting of livers provides overall benefit to the liver-recipient population.^11^ Despite these overall gains, relatively low numbers of livers are actually split in the UK.^12^ In the United States and other countries,^13^ rates of liver splitting are even lower. This may, in part, be due to concerns about the technical challenges and the negative outcomes with split liver transplantation but may also reflect the broader ethical concerns about the practice.

These ethical concerns need to be explored and addressed if they are posing an unnecessary barrier to further increasing the number of life-saving transplantations. Alternatively, if they represent a justifiable obstacle, the UK’s practice of splitting livers may need to be reviewed. An important aspect of exploring these concerns is gaining insight into how transplant staff and patients regard splitting livers, as they are the people likely to experience the consequences of the practice. In this article we present the findings of a qualitative study examining the views of senior transplant staff and liver transplant patients in the UK, and we use these results to inform a commentary on the ethical issues relating to split liver transplantation.

## Method

Qualitative interviews were undertaken with senior staff involved in liver transplantation and with liver transplant patients in the UK. Semistructured interviews were chosen to facilitate in-depth exploration of the views and values of participants: the flexibility to probe and prompt particular avenues of emerging thought offered by semistructured interviews enabled the collection of richer data. The overarching approach can be considered “empirical bioethics,” in which the data is gathered to inform, and provide a springboard for, ethical analysis.^14^ Allowing the interviewer to actively engage with the reasoning of participants via the introduction of counterfactuals and gentle disagreement helped us gain insight into not just participants’ views but also how these views were formed and reasoned. This method has already proved to be a useful way of exploring ethical issues.^15^

Purposive sampling was used to recruit liver transplant surgeons and physicians from five UK liver transplant centers. This population was most likely to be the best source of experience and understanding of split liver transplantation and its consequences and was therefore likely to hold the most informed views on the ethical dimensions of the practice. As staff working with pediatric patients may have different views from staff working with adult patients, two pediatric transplant centers were included.

Potential liver-recipient participants were selected from patients registered at the Queen Elizabeth Hospital Birmingham (QEHB). The QEHB is the liver transplant center for a large geographical area beyond Birmingham and so provides a diverse range of participants. Both pre- and posttransplant patients were identified, as it was anticipated that they may hold different views on splitting livers depending on their experiences of transplantation (e.g., whether they had received a split or whole liver, or whether they had experienced complications posttransplant).

The inclusion criteria limited participants to adult patients who (1) had had a liver transplant in the previous five years or (2) were on the liver transplant waiting list at the time of recruitment.

Interviews were transcribed verbatim then analyzed. Content analysis was used, using QSR nVivo software. The process commenced with immersion in the data by reading and rereading transcripts and listening to the audio recordings. Then followed relatively open coding of key concepts as they appeared in the transcripts, with codes then grouped into themes as analysis progressed. Analysis was an iterative process, and transcripts were recoded after analysis of other transcripts resulted in new codes or different uses of previous codes. Although the initial coding was open, due to the ethical nature of the overall project enquiry, there was a natural tendency to code according to ethical concepts.

A favorable ethical opinion was obtained from a National Health Service (NHS) research ethics committee and R and D permissions were obtained for each participating NHS trust.

## Results

Participant characteristics are summarized in Table 1. The key themes that emerged from the data can be summarized as follows:

1) Risk understanding of patients
2) Transplant optimism: quantity over quality
3) Sharing benefit: willingness for children to be prioritized

**Table 1.** Participant Characteristics

	Adult	Pediatric	Pretransplant	Posttransplant	Total
Liver staff (LS)	9	4			13
Liver patient (LP)			5	14	19

### Risk Understanding of Patients

The first theme to emerge from the data was poor understanding of risk on the part of patients. Transplant staff generally felt that patients did not have a detailed understanding of risk, but the staff took a relatively pragmatic attitude toward this, suggesting that it is not always necessary or beneficial for patients to understand everything. Some staff were concerned that giving patients more information might cause undue worry or result in patients making decisions that might be contrary to their best medical interests:

We will go some way towards trying to ensure that they [patients] have informed consent . . . but a lot of them don’t read it or take it in. And we certainly don’t give them . . . every minute detail, the figures and everything, because that would almost certainly result in people not doing operations which you know are actually very safe and that, actually, their overall general health would be less good because they wouldn’t have had the treatment that would have given them great benefit. (LS17)

Other staff accepted that there may be situations in which patients sign consent forms without fully understanding the risks involved but felt it important that attempts are made to explain risk as fully as possible:

I was generally taught that if you have a got a risk of something being greater than one in a thousand, then you should give full information to the patient. And so I think the answer is you should explain as much as possible and have the opportunity to answer questions, then if they don’t fully appreciate it but are happy to sign the consent now, I don’t think there’s anything further you can do. (LS2)

Patients often felt that they had a good understanding of the risks involved, but when prompted, it frequently transpired that their risk understanding was in very general terms. For example, the following patient greatly simplified the risks and benefits of receiving a split liver: “The way it was explained to me was that if I had the lesser liver, which I was destined for, it’s a little bit longer to get it kick-started into action, but, you know, people have successfully undergone that surgery” (LP26).

### Do-or-Die Optimism from Patients

It was clear that patients were generally aware of the dangers of remaining on the waiting list without a transplant, and this awareness led to many of them thinking that any liver offered to them would provide them with an opportunity for a better/longer life than not receiving a liver. This resulted in a high degree of optimism regarding transplantation, with transplants being viewed as an opportunity or a second chance that should not be missed. When asked whether they would have considered turning down a low-quality liver, this participant responded: “I don’t think I would have thought about it because if you’re in such a desperate situation that you need a new liver, I think whether it’s a high-quality one or not, it’s a second chance. And I think you’ve got to take a second chance” (LP5).

### More Opportunities Are Good

Patients’ relatively simplistic understanding of risk, and optimism toward transplantation, appeared to lead to many patient-participants feeling that quantity of transplants was more important than quality of transplants: “You know, you’re giving two people a chance instead of one. If they don’t work, it’s sod’s law, isn’t it? But I think everybody, as many people as possible, deserve a chance” (LP23).

Patient-participants showed a high degree of trust in their doctors’ judgement about the risks involved in transplantation, which is seemingly compatible with the view of staff-participants that staff are best positioned to make judgments about treatment and that providing too much information may disrupt the transplantation process. Many patient-participants stated that they had faith in transplant staff to not perform a transplant that was considered too risky. They tended to believe that they would not be offered a liver unless it was considered “good enough”: “I still don’t think they’d give me a risk . . . a liver which would be too high a risk because it’s a major operation and I don’t think they’re going to do an operation with something that’s too high a risk for it to be a success” (LP24).

This idea of maximizing the number of transplants was also fairly common among transplant staff, although there was obviously a much more nuanced understanding of risk within this group. Transplant staff were more likely to consider the quality of the offered organ and felt it important to maximize not simply the quantity of transplants but also the quantity of *reasonable*-quality transplants.

There are different ways to split livers and you can split a liver in such a way that you could give half of the liver to each adult, small adults, but the liver would have to be split slightly differently, and there’s a degree of expertise that’s needed for that. Again, I think that, in this era of organ shortage, anything that could be done to maximize reasonable-quality organs is of benefit. (LS13)

### Prioritizing Children

A further key theme to emerge from the data was support for prioritizing children and young adults. This view was prevalent within both staff and patient categories and contributed to a generally positive position on the splitting of livers.

Most patients felt it right for children to get some form of priority. A common justification for this was that children have lived less life than adults. This was rarely advanced into a full argument by participants, but there was a feeling that those who have lived less life ought to have some additional claims over those who have lived more: “Because I think it’s—I’m 55 now, I was 53, and children, they haven’t lived have they? And I would much rather a child’s life was saved. I mean I’ve got seven grandchildren, and if push came to shove I’d rather children be saved” (LP4).

The feeling among some transplant staff that prioritizing children was appropriate on the grounds that it is what society would want certainly resonated with the views of our patient-participants. One staff-participant went further and suggested that the public would find it completely unacceptable for there to be anything greater than a small percentage of children dying on the waiting list (although this was not specifically echoed by patient-participants):

Obviously, there will be children who die on the waiting list, that’s inevitable—but if it runs above a very, very, very low level, that would be deemed unacceptable. And the only way to ensure that you don’t have that sort of death rate on the waiting list is to keep the waiting times for all the children as low as possible. And that means, you know, they have access to all the pediatric organs, and they have access to all the healthy adult organs which can be split. (LS15)

Many adult patients were willing to carry the cost of helping children, even if that involved an increased risk of mortality or other less severe complications. Many participants in the study were middle aged or older and seemed content that they had already had a reasonable amount of good-quality life:

Any child. I think where it’s difficult, I mean I’m 60 when I had my transplant. And I knew there was a chance I was going to die. But I felt that at 60 well at least I’d had 60 good years of life. And for me, if it meant a split liver and it not working well, so be it, a child’s life might well have been saved. To me that’s a priority. And that wouldn’t just be my children or my grandchildren, it would be anybody’s. (LP7)

### Relatively Small Cost to Adults

Some transplant staff felt that the relative cost to adults is low in comparison to the gain for children, particularly because of the relatively small number of livers that are split.

They are all treated the same, but because we have our separate adult list, we don’t look at the children’s list and say, “All the kids should have preference to the adults,” but my feeling is that the children should have the benefit, particularly as we can, seemingly, offer them transplantation without too much detriment to the adults. The numbers of split livers are quite small, and the proportion of splits, compared with all the donors that adults are getting is, sort of, well under 5% would be my reckoning. (LS13)

### Priority for Younger People Generally: Not Just Children

Many participants felt that age-based priority should not be restricted to children and supported the idea that younger people more generally should receive priority over older people. This view was felt most strongly when there was a big age gap between two potential transplant candidates (a 20-year-old versus a 60-year-old), but many participants held the same view when the age gap decreased.

What you’re trying to do when you prioritize younger patients is to give them the opportunity to have a life. I think on those grounds it’s reasonable to prioritize, but where does that sliding scale stop, because I would argue that patients in their twenties probably should have priority over patients in their sixties. So I’m not sure that my argument holds purely for children. (LS29)

### Adults Feel Good about Sharing

Some adult patients stated that they felt good about the idea that part of the liver they received was also benefiting a child. This seemed to allow them to feel more positive about taking a scarce resource, as it appeared that they considered the decision to share a liver to be partly their decision to feel good about:

I thought “well as a human being I’ve done my bit but there’s a child here that needs it that’s had virtually no life at all.” And so . . . I felt that from my personal point of view very positive that I’d shared a liver. . . . And I found that very fulfilling that we’ve somehow got this other human that I don’t know, but we’re sharing something somebody else had to die to give us. That is almost . . . it forms the circle. (LP5)

### Split Livers Are Not Always the Worst Livers

A view prevalent among staff-participants was that, in the spectrum of livers that are offered for transplantation, split livers are certainly not the worst. “I think that you know, we can’t promise any adult now a good-quality organ, and split livers, along with other types of, what we classify as marginal donors, are really what we are now expecting to offer adults. It’s not very common that we can actually give them a perfect organ” (LS13).

## Discussion

### Limitations

This study presents the views of a relatively small number of liver transplant staff and adult patients. Despite the small sample size, the dataset obtained was nonetheless rich and contained a variety of views. The small size of the liver transplant community in the UK was a limiting factor—for instance, participant recruitment focused on senior liver transplant staff, as they were most likely to have experience and expertise in splitting livers, but with only a handful of liver transplant centers in the UK, the pool of potential participants was small. The sample size is, however, appropriate for the intended use of the data, which is to serve primarily as a springboard for consideration of ethical issues discussed by participants rather than as a means of adding overriding weight to particular arguments.

Patient-participants were recruited from the QEHB, a hospital that has a successful history of split liver activity: patients are educated about liver transplantation by transplant staff, and it is possible that positive staff attitudes toward the process may have influenced the views of patients. A further possible limitation is the age demographic of patient-participants: although age was not recorded, the majority of participants appeared middle aged: there were very few participants in their 20s, 30s, or 40s, and people of this age may have different views regarding prioritizing children or younger adults.

Although it was originally anticipated that recruitment would take place in each of the eight liver transplant centers in the UK, difficulties in obtaining R and D permissions within the time scale of the project meant that this was not possible. Although the five transplant centers used represented a mixture of adult and pediatric centers, it is possible that the centers that were not used may have encountered specific issues with split liver transplantation that were not raised within the sample.

### Risks Understanding

Liver transplantation is a complex procedure, and the risks associated with such a procedure are themselves complex: there are the risks of the operation itself, the postoperative risks, and the risks related to waiting longer for a liver, or not receiving a liver at all. Patient-centered care and involving patients in decisions about their treatment is considered important within the UK,^16^ but this approach may assume that patients have a reasonable understanding of the options available to them. Patients in our study, however, appeared to have limited understanding of the risks associated with transplantation, which may negatively impact their understanding of available options. Staff-participants generally felt that although patients do not understand risk well, staff do their best to explain risk at an appropriate level and with an appropriate level of detail for the patient. The limited risk understanding among patients corresponds with findings in other research.^17^ For some patient-participants, the apparent lack of understanding may have been related to the fact that they had their transplant some time ago and had simply forgotten the details, but the data were largely consistent between post- and pretransplant patients, and this explanation would not apply to these latter patients.

The lack of risk understanding is potentially concerning because it raises questions about the extent to which consent for transplantation can be considered informed, particularly with transplants that involve the more complex risks that split livers pose. There are specific guidelines for consent and organ transplantation. These state that information on the risks and benefits of transplantation should be given prior to a patient joining the waiting list.^18^ This includes information about specific types of livers—including split livers—that involve increased risk. Patient-participants in our study would have routinely received this information prior to being placed on the waiting list yet generally could not recall it in detail. Consent for transplantation is, as the NHSBT guidance notes, slightly peculiar insofar as it is given when a patient joins the waiting list, and with a significant time lag between being listed and transplanted. Over this period, the risks and benefits may change significantly, and so may a patient’s recall and understanding of them, so the NHSBT guidance suggests that, where possible, consent should be reaffirmed at the time of an organ being offered.^19^ A potential concern with this system is that this reaffirmation occurs against a backdrop of the need to minimize ischemic time for the donated liver. Additionally, the NHSBT guidance on consent for transplantation states that “where the proposed intervention is potentially life-saving and is the best life-saving therapeutic option available, many will not fully evaluate the risks of the procedure,”^20^ and this appeared to be the case with patient-participants in this study. This poor understanding of risk is not unique to transplantation,^21^ but the risks in transplantation may be particularly complex given the uncertain nature of liver supply, fluctuations in one’s health after joining the waiting list, and the various types of liver that one could be offered.

These concerns may be allayed somewhat by our data highlighting the role that transplant staff play in helping patients to reach decisions, with patients often willing to defer to the expertise of staff when making decisions about liver transplantation. Patient-participants did, therefore, feel that their risk understanding, combined with guidance from transplant staff, was sufficient for them to make informed decisions regarding transplantation. If patients are happy to defer to the expertise of transplant staff, particularly immediately pretransplant, when the patient is likely to be in a fast-moving situation without time to fully reflect on available options, then limited understanding of specific risks is not necessarily a significant problem. This may, however, raise the question of whether there are benefits to patients having detailed risk explained to them, either at the time of listing or again immediately pretransplant. Vulchev et al., in their paper on split liver transplantation, discuss the importance of explaining risk to patients, including the need for transplant surgeons to provide information on “the potential for increased morbidity, the possibility of further invasive interventions and the prospect of longer hospitalization,”^22^ and suggest that this should occur at the time of listing, or at least a substantial period before a patient is offered a transplant. Our study suggests that although this information may be explained to patients, it is not often fully understood or remembered by them to the extent that they can make fully informed decisions by themselves. Despite this, there may still be good reasons to provide information on risk to patients: for instance, the patients in our study *wanted* to know about the risks involved, even though they may not have understood or remembered it in minute detail. Additionally, some patients have better risk understanding than others, so taking a lowest-common-denominator approach may not be ideal. Some literature suggests that (not specifically transplant) patients often desire more information about risk than is currently provided,^23^ but this was not reflected by the findings from our study, in which patient-participants generally felt that they were given enough information.

One might worry that patients’ deference to transplant staff is problematic, but it is fair to assume that staff will understand the risks and benefits much better than patients, and it would be contrary to professional obligations for a patient to be offered a transplant that is contrary to their interests. There is, however, a possibility that staff may not always have an accurate understanding of their patients’ willingness to take risks, or how their patients balance competing risks and benefits, and such an understanding may be required to obtain a clear picture of a patient’s overall best interests. There may also be some tension for staff between protecting the interests of each individual patient and promoting equality and fairness in relation to the waiting list. For instance, splitting a liver may provide more overall benefit to the waiting lists but may not provide the maximum chance of good outcomes for an individual adult patient when compared to keeping the liver whole.

The current situation regarding understanding of risk and consent for transplantation appears workable but perhaps not ideal. Our patient-participants seem to want risk explained to them, and there is much literature suggesting that this is desirable.^24^ Despite wanting this, our patient-participants’ understanding and recall of risk did not appear sufficiently nuanced to result reliably in informed decisions. Patients were, however, willing to trust transplant staff to help them make decisions and to defer to their expertise. This may suggest that patients want to know about risk not so much to help them make decisions but to have a better understanding of their situation.

### Transplant Opportunities and Doing or Dying

We have discussed risk understanding in detail, because it is an important issue in itself, but also because it may explain the next theme emerging from our data. Simplistic understanding of the risks of transplantation appeared to lead to patients valuing the quantity of transplants over their quality. Some patient-participants, for instance, stated that all transplantation is risky and that there is risk involved in everything in life and concluded that taking additional risks is therefore not problematic—some participants specifically said, “You could get hit by a car/bus tomorrow.” Although it is true that there is some risk involved with most things, risks vary significantly in both their likelihood and consequences, so it does not follow that increased risk is of no consequence.

A prominent view from patients was that accepting a transplant was viewed as an opportunity to *avoid* the risks associated with waiting longer for a transplant or not receiving a transplant at all, and it is therefore better to have more of these opportunities available; transplantation was viewed as a do-or-die choice. This view, however, fails to take into consideration that not all opportunities are equal: some livers carry increased risks, and these risks will also depend on an individual patient’s condition. There are also many factors that may affect a patient’s probability of dying on the waiting list. It is too simplistic to say that it is better to have more livers available for transplant than fewer. Increasing the number of available livers by using incredibly poor-quality ones could make the choice more akin to an unacceptable die-or-die scenario, if the low-quality livers presented sufficient risks to recipients. A more justifiable claim is that it is better to have more sufficient-quality transplants than fewer (and this was the qualification that staff-participants tended to add). This leaves open the matter of what “sufficient quality” might mean. A reasonable starting point is that the liver should be expected to provide benefit to the particular patient to whom it is offered, as a patient should not be offered a liver with no expected benefit. Staff-participants often emphasized that the chronic shortage of livers means that it is not possible to give everyone a good-quality liver, and that in the overall hierarchy of livers, split livers can often be considered relatively good (although they have been split, they are from young and previously healthy donation-after-brain-death donors with limited time spent in the ICU).

Splitting livers allows for more transplants, but the risks of morbidity and mortality may be greater than with the equivalent whole liver. This method of increasing the number of transplants available decreases the risk of dying on the waiting list (particularly for children) but increases the risk of posttransplant complications. These risks have to be carefully balanced (and the improved results^25^ with split livers suggests that the practice may provide overall benefit), but adult patients are exposed to more risk than they would be if the same adult livers were kept whole. Increased risks are something that patients might ordinarily tend to avoid, but the next theme—prioritizing children—suggests that adult patients may have some reasons to accept increased risks.

### Prioritizing Children and Sharing Benefit

The majority of participants, both patients and staff, thought it appropriate that children should be prioritized for transplantation. That adult patient-participants were generally happy for children to be advantaged within liver transplantation would suggest that the current situation in which children have shorter waiting times than adults is not considered objectionable, despite the potential for theoretical concerns about age discrimination or injustice. Support for prioritizing children was observed in the United States by Barshes et al., who found that 62.5% of participants felt that children should receive priority (18% felt neutral, and 18% disagreed).^26^

Many participants justified differential treatment on the grounds that children have lived less life, which may reflect a fair innings–style argument.^27^ This argument claims that there is a reasonable amount of life that a person can expect to have, and that those who have not had this amount of life should receive priority over those who have, when it comes to the allocation of resources that may sustain life.^28^ Therefore, those who are over the age of, for instance, the biblical threescore years and ten should receive lower priority than children. Although this discriminates against older people, it is claimed that this is fair because they have already had something that younger people are lacking, namely, a fair innings. A view shared by several participants, which again points toward a fair innings–style argument, was that younger people, and not specifically children, should be prioritized over older people (for instance, a 25-year-old should be prioritized over a 50-year-old).

Importantly, support from adult patient-participants for prioritizing children was reinforced by a willingness to sacrifice some of their own outcomes, or to expose themselves to greater risks, for the benefit of children or younger people. This is similar to findings in the Barshes study, which reported that 90% of participants would be willing to “share” their transplant even if it resulted in a shorter lifetime for them.^29^ It should be noted that most participants in our study were middle aged and so may have felt that they had already had, or were close to having, their fair innings.

Although most participants supported the *prioritization* of children, this is something of a red herring when it comes to justifying the splitting of livers. Without any form of liver splitting, children would be severely disadvantaged in comparison to adults in terms of waiting times and waiting list mortality. Before liver splitting in its current form was developed, a technique was available that reduced the size of an adult liver to something suitable for a pediatric recipient but discarded the remainder of the liver.^30^ This practice could be justified on the grounds of equality of access to the resource: although the adult liver-patient population had fewer livers as a result, the pediatric population had a corresponding gain, which resulted in increased equality. Although equality is only one consideration, it is prima facie desirable to have more equality than less. The current form of liver splitting can be framed as having improved on a justifiable technique of cutting down livers so that it is no longer necessary to divert livers from adults to children: now adults can also receive benefit, which should provide greater overall utility. A practice that was once justified on the grounds of equalizing the opportunities for adults and children can now also be justified by increased overall utility. The “problem” now faced is that there is, in the UK at least, a new inequality between adults and children. As a result of split liver transplantation, children’s average waiting time is now *less* than adults’, but this inequality can only be addressed at present by leveling down, because the benefits of the graft resulting from a split liver currently destined for a child cannot be effectively diverted to the adult list. The results from this qualitative study, however, suggest that adult patients and transplant staff generally feel that privileging the interests of children over adults is acceptable.

## Conclusions

Patients and staff were generally supportive of split liver transplantation and felt that, given the shortage of transplantable livers, efforts should be made to increase the number of opportunities for patients to receive a transplant. Splitting livers achieves this goal, but at some cost to the adult recipients of split livers. Most patient-participants in this study were, in principle, willing to bear this cost, when children or younger adults were the likely beneficiaries. It was apparent, however, that they were not often fully aware of the extent of the additional risks to which they may be exposed. The study has highlighted that even if patients have risk explained to them, their understanding may not always be detailed, and they rely heavily on transplant staff to help them make decisions about transplants: and our patient-participants did not find this to be unacceptable. It is often assumed that an understanding of risk and benefit is an important part of informed consent for treatment,^31^ but our study suggests that deference to staff expertise rather than patient understanding of risk and benefit is what guides treatment decisions in many cases. This is not necessarily problematic but may fuel further debate over how and when information about risks and benefits should be explained to transplant patients.

Splitting livers in the UK has mostly resolved the initial problem that justified its introduction: children now tend to have relatively short waiting times for liver transplants, and one of the factors limiting the number of livers that can be split is the availability of suitable pediatric recipients. Although there are technical hurdles, some promising results have been obtained using full left–right splits to enable two adults to benefit from the same donated liver.^32^ The views of participants in this study would support this as an acceptable way to increase the number of people receiving transplants if there were no suitable pediatric recipients available. Achieving consistently acceptable results with full left–right splits may take time, if indeed it is ever possible, but in the meantime the current practice of splitting livers for an adult and a child appears to be an acceptable way to increase the number of patients receiving transplants.

## Notes

1. NHS Blood and Transplant. Latest statistics; available at http://www.organdonation.nhs.uk/statistics/downloads/weekly_stats.pdf (last accessed 1 Apr 2014).

2. See note 1, NHS Blood and Transplant.

3. NHS Blood and Transplant. Liver transplant activity report; available at http://www.organdonation.nhs.uk/statistics/transplant_activity_report/current_activity_reports/ukt/liver_activity.pdf (last accessed 1 Apr 2014).

4. See note 3, NHS Blood and Transplant.

5. EmreS, UmmanV. Split liver transplantation: An overview. Transplantation Proceedings 2011;43(3): 884–7.2148662010.1016/j.transproceed.2011.02.036

6. OtteJB, de Ville de GoyetJ, RedingR, Van ObberghL, VeyckemansF, CarlierMA, et al Pediatric liver transplantation: From the full-size liver graft to reduced, split, and living related liver transplantation. Pediatric Surgery International 1998;13(5–6):308–18.963960610.1007/s003830050328

7. BusuttilR, GossJA. Split liver transplantation. Annals of Surgery 1999;229(3):313–21.1007704210.1097/00000658-199903000-00003PMC1191695

8. EmondJC, FreemanRB, RenzJF, YersizH, RogiersX, BusuttilRW. Optimizing the use of donated cadaver livers: Analysis and policy development to increase the application of split-liver transplantation. Liver Transplantation 2002;8(10):863–72.1236042610.1053/jlts.2002.34639

9. Braata AE, Bloka JJ, Putter H, Adam R, Burroughs AK, Rahmele AO, et al. The Eurotransplant Donor Risk Index in liver transplantation: ET-DRI. *American Journal of Transplantation* 2012;12:2789–96.

10. RenzJF, YersizH, ReichertPR, HisatakeGM, FarmerDG, EmondJC, et al Split liver transplantation: A review. American Journal of Transplantation 2003;3(11):1323–35;1452559110.1046/j.1600-6135.2003.00254.x MerionRM, RushSH, DykstraDM, GoodrichN, FreemanRBJr, WolfeRA. Predicted lifetimes for adult and pediatric split liver versus adult whole liver transplant recipients. American Journal of Transplantation 2004;4(11):1792–7.1547647810.1111/j.1600-6143.2004.00594.x

11. See note 10, Merion et al. 2004.

12. NHS Blood and Transplant Liver Advisory Group. Liver splitting activity report; available at http://www.odt.nhs.uk/pdf/advisory_group_papers/LAG/Liver_splitting_activity_report_may2013.pdf (last accessed 29 May 2014).

13. Shrem H, Kleine M, Lankisch TO, Kalternborn A, Kousoulas L, Zachau L, et al. Long-term results after adult ex situ split liver transplantation since its introduction in 1987. *World Journal of Surgery* 2014; available at http://link.springer.com/article/10.1007/s00268-013-2444-4 (last accessed 1 Apr 2014).

14. BorryP, SchotsmansP, DierickxK. The birth of the empirical turn in bioethics. Bioethics 2005;19(1):49–71.1581297210.1111/j.1467-8519.2005.00424.x

15. For example, Alderson P, Farsides B, Williams C. Examining ethics in practice: Health service professionals’ evaluations of in-hospital ethics seminars. *Nursing Ethics* 2002;9:508–21.

16. Bridson J. Making consent patient centred. *British Medical Journal* 2003;327:1159.

17. LloydAJ. The extent of patients’ understanding of the risks of treatments. Quality in Health Care 2001;10(1 Suppl):14–18.10.1136/qhc.0100014..PMC176573411533432

18. NHS Blood and Transplant. Guidelines for consent for solid organ transplantation in adults; available at http://www.nhsbt.nhs.uk/download/guidelines_for_consent.pdf (last accessed 1 Apr 2014).

19. See note 18, NHS Blood and Transplant.

20. See note 18, NHS Blood and Transplant, at 9.

21. See note 17, Lloyd 2001.

22. Vulchev A, Roberts JP, Stock PG. Ethical issues in split versus whole liver transplantation. *American Journal of Transplantation* 2004;4(11):1737–40, at 1740.

23. EdwardsA, ElwynG, MulleyA. Explaining risks: Turning numerical data into meaningful pictures. British Medical Journal 2002;324(7341):827–30.1193477710.1136/bmj.324.7341.827PMC1122766

24. PalingJ. Strategies to help patients understand risk. British Medical Journal 2003;327(7417):745–8.1451248910.1136/bmj.327.7417.745PMC200818

25. See note 12, NHS Blood and Transplant Liver Advisory Group.

26. Barshes N, Lee TC, Udell IW, O’Mahoney CA, Carter BA, Karpen SJ, et al. Adult liver transplant candidate attitudes toward graft sharing are not obstacles to split liver transplantation. *American Journal of Transplantation* 2005;5(8):2047–51.

27. HarrisJ. Does justice require that we be ageist? Bioethics 1994;8(1):74–83.1165737610.1111/j.1467-8519.1994.tb00242.x

28. Harris J. *The Value of Life, an Introduction to Medical Ethics*. London: Routledge & Keegan Paul; 1970.

29. See note 26, Barshes et al. 2005, at 2048.

30. EmondJC, WhitingtonPF, ThistlethwaiteJR, AlonsoEM, BroelschCE. Reduced-size orthotopic liver transplantation: Use in the management of children with chronic liver disease. Hepatology 1989;10:867–72.280716810.1002/hep.1840100520

31. General Medical Council. Consent: Patients and doctors making decisions together; available at http://www.gmc-uk.org/static/documents/content/GMC_Consent_0513_Revised.pdf (last accessed 1 Apr 2014).

32. Zambelli M, Andorno E, De Carlis L, Rossi G, Cillo U, De Feo T, et al. Full-right-full-left split liver transplantation: The retrospective analysis of an early multicenter experience including graft sharing. *American Journal of Transplantation* 2012;12(8):2198–210.

